# Developmental Gains in Visuospatial Memory Predict Gains in Mathematics Achievement

**DOI:** 10.1371/journal.pone.0070160

**Published:** 2013-07-31

**Authors:** Yaoran Li, David C. Geary

**Affiliations:** 1 Department of Educational, School, and Counseling Psychology, University of Missouri, Columbia, Missouri, United States of America; 2 Department of Psychological Sciences, University of Missouri, Columbia, Missouri, United States of America; 3 Interdisciplinary Neuroscience Program, University of Missouri, Columbia, Missouri, United States of America; University of Leicester, United Kingdom

## Abstract

Visuospatial competencies are related to performance in mathematical domains in adulthood, but are not consistently related to mathematics achievement in children. We confirmed the latter for first graders and demonstrated that children who show above average first-to-fifth grade *gains* in visuospatial memory have an advantage over other children in mathematics. The study involved the assessment of the mathematics and reading achievement of 177 children in kindergarten to fifth grade, inclusive, and their working memory capacity and processing speed in first and fifth grade. Intelligence was assessed in first grade and their second to fourth grade teachers reported on their in-class attentive behavior. Developmental gains in visuospatial memory span (d = 2.4) were larger than gains in the capacity of the central executive (d = 1.6) that in turn were larger than gains in phonological memory span (d = 1.1). First to fifth grade gains in visuospatial memory and in speed of numeral processing predicted end of fifth grade mathematics achievement, as did first grade central executive scores, intelligence, and in-class attentive behavior. The results suggest there are important individual differences in the rate of growth of visuospatial memory during childhood and that these differences become increasingly important for mathematics learning.

## Introduction

Children and adolescents with a high working memory capacity consistently outperform their lower capacity peers on mathematical cognition and achievement measures, but the relative contributions of the different components of working memory to these differences are not fully understood [1]–[6]. The majority of studies that have examined the relation between different aspects of working memory and mathematical performance have followed Baddeley and Hitch’s [7] three component framework; specifically, the central executive and the phonological and visuospatial short term memory systems. The central executive includes the attentional and inhibitory control mechanisms that enable the mental representation of multiple pieces of phonological or visuospatial information in working memory and the simultaneous mental manipulation of this information. Of these three components, competencies captured by the central executive are the most consistently related to mathematical learning and achievement from preschool to adolescence [1], [6], [8]–[11].

The contributions of phonological and visuospatial memory to mathematics performance, in contrast, are typically reduced in magnitude and often eliminated when performance on measures of the central executive and other factors (e.g., intelligence) are controlled [12]–[15]. When independent contributions are found, the importance of phonological and visuospatial memory varies with the content and complexity of the mathematics being processed [1], [16]–[19]. Phonological memory has been found to contribute to the encoding and processing of number words and numerals and to processes that involve them, such as using counting procedures to solve arithmetic problems (e.g., counting ‘five, six, seven, eight’ to solve 5+3) or retrieving arithmetic facts from long-term memory [16]–[18], [20]–[21]. Phonological memory is sometimes but not always found to be related to skill at solving mathematical word problems (i.e., problems that are stated in written form and have to be translated into a mathematical problem to be solved), presumably because phonological skills contribute to the reading-related components of word problems [15], [22].

Visuospatial memory appears to be important for some aspects of mathematics, as in mentally visualizing and representing quantities on the number line [18], [23] and contributes to the solving of word problems [16], [22]. The latter effect is likely related to ease of constructing diagrams to represent quantitative relations in the word problems. However, the strength of the relations between visuospatial memory and mathematics performance varies with age or school grade and when found are typically smaller than the contribution of the central executive [15], [24]–[25]. The inconsistent relation between visuospatial memory and mathematics performance in these developmental studies is particularly surprising (see [26]), given the more consistent relation between spatial ability and mathematics performance [27]–[31] and accomplishment in mathematics-intensive occupations in adulthood [32]–[33]. One potential reason for the stronger relation between visuospatial ability and mathematics performance in older than younger samples is differences in the mathematical competencies assessed across studies [34].

Another potential factor may be maturational change in the working memory system itself [35]. During the preschool years, individual differences in working memory are best described by a single attentional or effortful control competence [36]. The differentiation of the working memory system into reliably assessed central executive, phonological memory, and visuospatial memory systems appears to occur by about five years of age [37]–[38]. In a large cross-sectional study, Gathercole et al. found similar rates of growth in the three components of working memory from four-five years of age to fourteen-fifteen years of age. However, for the same tasks we used in the current study and for the ages we assessed, Gathercole et al. found the largest gains for visuospatial memory, followed by the central executive, and phonological memory. Several longitudinal studies also suggest differences in the rate of gain in the three components of working memory. Engel de Abreu et al. [35] found larger kindergarten to second grade gains in the central executive (ds = 2.2–2.5) than phonological memory (d = 0.6–1.3), and Andersson [16] found slightly larger third to fifth grade gains in visuospatial memory (*d* = 1.0) than the central executive (d = 0.7).

In other words, differential gains in the central executive, phonological memory, and visuospatial memory could result in across-grade changes in the relative contribution of these components of working memory to mathematics performance. These gains could be related to maturation of the supporting brain systems or to improvements in the ability to strategically use these competencies during problem solving, for instance functionally improving memory performance by rehearsing the to-be-remembered information [39]–[40]. Whatever the reason, there is some evidence to suggest that across-grade change in mathematical performance may track across-grade change in working memory competencies. Swanson [41] assessed working memory development from first to third grade as related to competence at solving arithmetical word problems. First grade performance on measures of the central executive and phonological memory predicted skill at solving word problems in third grade. First to third grade growth in problem solving ability was related to *gains* in the central executive, but not to gains in phonological or visuospatial memory. Van der Ven et al. [11] found that performance on first grade measures of the central executive predicted mathematics achievement but first to second grade gains in the central executive did not. The latter result is difficult to interpret because the one year time span resulted in less opportunity for growth in the central executive than in Swanson’s study.

In any case, the question of how growth in the working memory system affects mathematics achievement above and beyond the relation between school-entry working memory and later achievement is largely unexplored. Differences in the types of visuospatial competencies assessed across studies may also contribute to the inconsistent relation between visuospatial abilities and mathematics performance [34]. For a sample of adolescents, Kyttälä and Lehto [5] found that the relation between visuospatial ability and mathematics performance, controlling for fluid intelligence, varied with whether the visuospatial task required simultaneous (e.g., maze memory, see Methods), sequential (e.g., block recall, Methods), or active (e.g., three dimensional mental rotation) processing. Memory for simultaneously presented visuospatial information predicted overall performance on a standardized mathematics achievement test and especially performance on a subset of orally presented word problems, whereas sequential processing made unique contributions to performance on written word problems and active processing to geometry.

### Current Study

We were particularly interested in whether the strength of the relation between visuospatial memory and mathematics achievement increased with development [24]. We assessed this by examining the relation between the three components of working memory and mathematics achievement in fifth grade, controlling for the relation between first grade working memory capacity and grade-related changes in mathematics achievement and controlling for other factors that are related to mathematics learning; specifically, intelligence [42], in-class attentive behavior [13], [43] and processing speed [44]. In effect, the control of first grade working memory scores allowed us to assess whether first to fifth grade gains in working memory contributed to mathematics achievement in fifth grade. We also examined whether simultaneous visuospatial memory was a better predictor of overall mathematics achievement than sequential visuospatial memory, as found by Kyttälä and Lehto [5]; unfortunately, our study did not include a measure of active visuospatial memory. We also analyzed these relations for word reading achievement to provide a discriminative contrast to the analyses of mathematics achievement.

## Materials and Methods

### Ethics Statement

The study was reviewed and approved by the Institutional Review Board of the University of Missouri. Written consent was obtained from all parents, and all participants provided verbal assent for all assessments.

### Participants

The participants were children from a prospective study of mathematical development and risk of learning disability who had completed extensive working memory assessments in both first and fifth grades (see [8], [17], [45]). Two hundred sixty eight children completed the working memory assessment in first grade and 187 of these children completed the same assessment in fifth grade. Ten of these 187 children were dropped because achievement data were missing for one or more grades. Thus, the final sample included 177 (82 boys) children.

At the end of first grade, the mean intelligence of these 177 children was average (M = 102, SD = 14) based on the Wechsler Abbreviated Scale of Intelligence (WASI; [46]). Their kindergarten (M = 103, SD = 13) and fifth grade (M = 99, SD = 17) mathematics achievement were average, and their word reading achievement was above average in kindergarten (M = 113, SD = 14) and average in fifth grade (M = 104, SD = 11). The mean respective ages at the kindergarten and fifth grade achievement assessments were 6 years, 2 months (SD = 4 months) and 11 years, one month (SD = 4 months). Their ages were 7 years, 0 months (SD = 6 months) and 10 years, 8 months (SD = 5 months) for the first and fifth grade working memory assessments, respectively. Seventy-four percent the sample was White, and the remaining children were Black, Asian, or of mixed race.

In relation to the 177 children in the final sample, the 91 children who were dropped had lower intelligence (M = 94, SD = 15), F(1,263) = 16.02, p<.0001, and mathematics achievement (M = 99, SD = 14), F(1,266) = 6.08, p = .0143, scores in first grade, although both scores were in the average range for the dropped sample. There was also a trend for this group to have lower span scores for the central executive (M = 2.00, SD = .59), F(1,266) = 3.79, p = .0526, and visuospatial memory (M = 2.62, SD = .75), F(1,266) = 3.86, p = .0506, in first grade. There were no group differences for first grade reading achievement (M = 110, SD = 15 for the dropped group), p = .1045, or phonological memory span (M = 3.28, SD = .68 for the dropped group). One consequence of these group differences is a potential underestimation of the relation between working memory and mathematics achievement.

### Standardized Measures

#### Intelligence

The Vocabulary and Matrix Reasoning subtests of the WASI were administered and used to estimate intelligence based on procedures outlined in the manual [46]. For this nationally (U.S.) normed test, the mean score is 100 and the SD is 15.

#### Achievement

Mathematics and reading achievement were assessed using the nationally (U.S.) normed Numerical Operations and Word Reading subtests from the Wechsler Individual Achievement Test-II-Abbreviated [47], respectively. The easier Numerical Operations items assess number discrimination, rote counting, number production, and basic addition and subtraction. More difficult items include multi-digit addition and subtraction, multiplication and division, and rational number problems as well as simple algebra and geometry problems solved with pencil-and-paper. Based on the mean number of correctly solved problems (±1 SD) at the end of fifth grade, the majority of children could solve multicolumn addition and subtraction problems (e.g., 894+239), simple whole number multiplication and division problems (e.g., 4×7; 15 ÷ 3), and addition and subtraction of simple rational numbers (e.g., 3/4–1/3;.7+.4).

The easier Word Reading items require matching and identifying letters, rhyming, beginning and ending sounds, and phoneme blending. The more difficult items assess accuracy of reading increasingly difficult words.

### Working Memory

The Working Memory Test Battery for Children (WMTB-C; [48]) is a standardized and normed (U.K.) test of the three core components of working memory. The test consists of nine subtests that assess the central executive (three subtests, α = .75,.68 for first and fifth grades, respectively), phonological memory span (four subtests, α = .78,.77), and visuospatial memory span (two subtests, α = .55,.57). All of the subtests have six items at each span level. Across subtests, the span levels range from one to six to one to nine. Passing four items at one level moves the child to the next. At each span level, the number of items to be remembered is increased by one. We used total number of correct items in the analyses because these are more reliable than span scores.

#### Central executive

The three measures require the child to maintain one set of information in mind, while processing another set of information. Listening Recall requires the child to determine if a sentence is true or false, and then recall the last word in a series of sentences. Counting Recall requires the child to count a set of 4, 5, 6, or 7 dots on a card, and then to recall the number of counted dots at the end of a series of cards. Backward Digit Recall is a standard backward digit span.

#### Phonological memory

The four measures assess the child’s verbatim memory for phonological sounds. Digit Recall, Word List Recall, and Nonword List Recall are span tasks with differing content stimuli; the child’s task is to repeat words spoken by the experimenter in the same order as presented. In the Word List Matching task, a series of words, beginning with two words, is presented to the child. The same words, but possibly in a different order, are then presented again; the task is to determine if the second list is in the same or different order than the first list.

#### Visuospatial memory

Block Recall assesses sequential visuospatial memory as assessed by Kyttälä and Lehto [5] and consists of a board with nine raised blocks in what appears to the child as a “random” arrangement. The blocks have numbers on one side that can only be seen from the experimenter’s perspective. The experimenter taps a block (or series of blocks), and the child’s task is to duplicate the experimenter’s sequence. In the Mazes Memory task, which assesses simultaneous visuospatial memory, the child is presented a maze with more than one solution, and a picture of an identical maze with a path drawn for one solution. The picture is removed and the child’s task is to duplicate the path in the response booklet.

### Processing Speed

Speed of encoding and articulating numerals and letters was assessed using the rapid automatized naming (RAN) task [49]. Five letters or numerals are first presented to determine if the child can read the stimuli correctly. The child is then presented with a 5×10 matrix of incidences of these letters or numerals and is asked to name them as quickly as possible without making any mistakes [50]. Reaction time (RT in sec) is measured using a stopwatch.

### In-Class Attentive Behavior

The Strength and Weaknesses of ADHD–symptoms and normal-behavior (SWAN) was used as the measure of in-class attentive behavior [51]. The measure includes items that assess attentional deficits and hyperactivity but the scores are normally distributed, based on the behavior of a typical child in the classroom. The nine item (e.g., “Gives close attention to detail and avoids careless mistakes”) measure was distributed to the children’s second, third, and fourth grade teachers who were asked to rate the behavior of the child relative to other children of the same age on a 1 (far below) to 7 (far above) scale. Scores across grades were highly correlated, rs = .71 to.75 (ps<.0001), and thus for children with multiple ratings a composite was created using their mean (α = .88). At least one rating was available for 149 children, and missing scores for the remaining 28 children were estimated (maximum likelihood estimates with 5 imputations) using the multiple imputations program of SAS [52].

### Procedure

Achievement tests were administered every spring beginning in kindergarten, the WASI [46] in the spring of first grade, and the RAN every fall beginning in first grade. The majority of children were tested at their school site, and occasionally on the university campus or in a mobile testing van. Testing in the van occurred for children who had moved out of the school district and for administration of the WMTB-C (e.g., after school). The assessments required between 40 and 60 min.

### Statistical Analysis

Kindergarten to fifth grade raw scores from the achievement tests were analyzed using multilevel modeling; specifically, PROC MIXED [52]. Linear slopes for grade and intercept values were random effects, with grade coded sequentially from 0 to 5 for fifth grade to kindergarten, respectively. Intercept values thus represent achievement at the end of fifth grade. Predictors of fifth grade achievement (intercept) and grade-related changes in achievement (slope) were intelligence, in-class attentive behavior, first-grade working memory, and RAN RTs. We used numeral RTs to predict Numerical Operations scores and letter RTs to predict Word Reading scores. We separated these because speed of encoding Arabic numerals and articulating number words are basic processes that may affect children’s early skill at solving mathematics problems [53] and speed of articulating letters is related to children’s early competence at reading words [49]. The relation between across-grade gains in working memory and RAN RTs and fifth grade achievement were estimated with inclusion of the corresponding fifth grade measures. Because sex differences are often found on mathematics and reading achievement tests [54], a boy (coded 1) versus girl (coded 0) contrast was included in the mixed models. Except for achievement and the sex contrast, all variables were standardized using PROC STANDARD [52] with M = 0 and SD = 1.

## Results

### First-to-Fifth Grade Gains in Working Memory and Processing Speed

Total scores are the better measure, psychometrically, than span scores, but span scores provide a more readily interpretable estimate of the child’s functional skills. For instance, a span score of 2 for the central executive means that the child can reliably hold two pieces of information in working memory while processing other information. A phonological memory span of 3 means that the child can accurately recall three phonological pieces of information, such as words, immediately after they have been presented. For this reason, we report span scores in Table 1; the effect sizes for span (d in Table 1) and total correct (d_2_) are essentially the same. To assess the magnitude of change, scores for each component were combined across grades and standardized. Scores increased from first to fifth grade for all three components of working memory (ps = .0001). The grade by component interactions in repeated measures analyses of variance indicated that visuospatial memory increased more than the capacity of the central executive, F(1,352) = 3.63, p = .0575, and phonological memory, F(1,352) = 22.87, p = .0001, and central executive capacity increased more than phonological memory, F(1,352) = 15.07, p = .0001. RTs decreased (i.e., they were faster) significantly across grades for numerals and letters (ps = .0001); RTs were faster for letters than numerals in first, F(1,176) = 9.88, p = .002, and fifth, F(1,176) = 5.36, p = .0217, grade, but the magnitude of the difference decreased across grades, F(1,176) = 5.06, p = .0257.

**Table 1:** Working Memory Spans and Speed of Processing.

	First Grade	Fifth Grade	Gains			
	M (SD)	M (SD)	M	SD	d	d_2_
Central executive	2.15 (.55)	3.04 (.56)	0.89	0.51	1.6	1.7
Phonological memory	3.30 (.62)	3.99 (.63)	0.70	0.54	1.1	1.1
Visuospatial memory	2.79 (.63)	4.21 (.82)	1.42	0.76	2.3	2.4
Mazes Memory	2.10 (.78)	4.13 (1.13)	2.03	1.14	2.6	2.8
Block Recall	3.48 (.80)	4.29 (.85)	0.81	0.92	1.0	1.2
Numeral RAN RT	42.2 (12.1)	25.8 (5.3)	16.5	10.5	1.4	–
Letter RAN RT	40.2 (11.8)	25.2 (5.2)	15.0	10.4	1.3	–

Note: Span scores are reported for the working memory measures and reaction time (RT) in seconds for the rapid automatized naming (RAN) measures. d = effect size, (|M_5_–M_1_|)/SD_1_, where M_5_ and M_1_ are the respective means for fifth and first grade, and SD_1_ is the standard deviation for first grade. d_2_ = effect size based on total correct. In first and fifth grades respectively, the central executive was correlated with phonological (rs = .51,.60) and visuospatial (r = .48,.57) memory spans (ps = .0001), which in turn were correlated with each other (r = .25,.45, ps = .0008).

If the gains in phonological and visuospatial memory are related to improvements in the strategic use of these systems, for instance by using rehearsal to help remember the presented information [39]–[40], [55], then controlling for capacity of the central executive, which supports strategy use, should reduce or eliminate these gains. Controlling for capacity of the central executive in first and fifth grades, reduced the phonological memory gains to non-significance (p = .1560, d = .15), and gains for visuospatial memory were reduced but still substantial (p = .0001, d = 1.06). The corresponding effects also remained substantial for Mazes Memory (p = .0001, d = 1.01), but were reduced for Block Recall (p = .0053, d = 0.31). Gains in visuospatial memory were further reduced but remained significant with control of the interaction between the central executive, phonological memory, and time (i.e., changes from first to fifth grade) (p = .0001, d = .89); adding numeral and letter RTs, intelligence, and in-class attentive behavior and their interactions with time as further controls did not affect these results (p = .0001, d = .88). The corresponding effect size was somewhat larger for Mazes Memory (p = .0001, d = .99) and considerably lower for Block Recall (p = .0299, d = .29).

### Working Memory Gains and Achievement

Correlations among the predictor variables and fifth grade achievement scores are shown in Table 2. The girl vs. boy on intercept contrast effects in the top section of Table 3 show sex differences in fifth grade achievement, whereas the girl vs. boy on slope effect tests for sex differences in earlier grades relative to fifth grade. The predictor on intercept effects in the middle portion of Table 3 show the relation between first grade working memory, intelligence, and processing speed, as well as in-class attentive behavior, and end of fifth grade achievement. The predictor on slope effects show the relation between these variables and across-grade changes in achievement, i.e., how the effects differ in earlier grades relative to fifth grade. The predictor on intercept effects in the bottom section of Table 3 show the relation between fifth grade working memory and processing speed and fifth grade achievement, controlling for first grade scores on these same measures, as well as intelligence and in-class attentive behavior.

**Table 2:** Correlations Among Predictors and Fifth Grade Achievement Scores.

Variable	1	2	3	4	5	6	7	8	9	10	11	12	13	14	15	16	17
1: Intelligence	1.00																
2.In-class attentive behavior	.24**	1.00															
3∶1^st^ Grade CE	.40**	.57**	1.00														
4∶1^st^ Grade PM	.51**	.35**	.60**	1.00													
5∶1^st^ Grade VSM	.32**	.44**	.57**	.45**	1.00												
6∶1^st^ Grade BR	.23**	.44**	.50**	.41**	.83**	1.00											
7∶1^st^ Grade MM	.29**	.28**	.45**	.34**	.83**	.38**	1.00										
8∶1^st^ Grade Number RT	−.25**	−.44**	−.50**	−.41**	−.37**	−.33**	−.29**	1.00									
9∶1^st^ Grade Letter RT	−.29**	−.42**	−.43**	−.37**	−.32**	−.31**	−.22**	.74**	1.00								
10∶5^th^ Grade CE	.43**	.53**	.60**	.51**	.43**	.40**	.31**	−.44**	−.47**	1.00							
11∶5^th^ Grade PM	.30**	.26**	.47**	.71**	.28**	.24**	.22**	−.33**	−.31**	.51**	1.00						
12∶5^th^ Grade VSM	.25**	.45**	.45**	.27**	.53**	.46**	.43**	−.34**	−.33**	.48**	.25**	1.00					
13∶5^th^ Grade BR	.20**	.38**	.37**	.25**	.44**	.42**	.30**	−.22**	−.28**	.48**	.30**	.77**	1.00				
14∶5^th^ Grade MM	.22**	.38**	.38**	.21**	.46**	.36**	.41**	−.34**	−.28**	.35**	.15*	.90**	.40**	1.00			
15∶5^th^ Grade Number RT	−.06	−.30**	−.30**	−.15*	−.22**	−.19**	−.17*	.50**	.43**	−.28**	−.23**	−.27**	−.29**	−.19*	1.00		
16∶5^th^ Grade Letter RT	−.21**	−.29**	−.28**	−.23**	−.16**	−.13	−.14	.50**	.47**	−.34**	−.28**	−.26**	−.24**	−.21**	.83**	1.00	
17∶5^th^ Grade NO	.39**	.50**	.54**	.40**	.38**	.32**	.32**	−.38**	−.33**	.50**	.27**	.38**	.30**	.33**	−.19**	−.20**	1.00
18∶5^th^ Grade WR	.50**	.38**	.48**	.51**	.26**	.20**	.23*	−.52**	−.42**	.52**	.44**	.36**	.29**	.31**	−.32**	−.42**	.52**

Note: CE = Central Executive; PM = Phonological Memory; VSM = Visuospatial Memory; BR = Block Recall; MM = Mazes Memory; NO = Numerical Operations; WR = Word Reading.

** p<.01;

* p<.05.

**Table 3:** Results for Mixed Models Predicting Growth in Academic Achievement.

Predictor	Numerical Operations		Word Reading	
	Estimate (se)	p	Estimate (se)	p
Intercept	26.70(.39)	.0001	112.38 (.87)	.0001
Grade	−3.86(.09)	.0001	−11.06(.23)	.0001
Girl vs. Boy on intercept	−2.31(.56)	.0001	−1.16(1.25)	.3536
Girl vs. Boy on slope	.36(.13)	.0046	.85(.32)	.0084
**First Grade Working Memory, Intelligence, Attentive Behavior, and RAN RT Predictors of Achievement**				
Intelligence on intercept	.86(.31)	.0063	1.90(.71)	.0074
In-class attentive behavior on intercept	1.64(.34)	.0001	.71(.78)	.3609
1^st^ grade central executive on intercept	1.36(.40)	.0007	.68(.88)	.4381
1^st^ grade phonological memory span on intercept	−.02(.37)	.9530	1.90(.94)	.0433
1^st^ grade visuospatial memory span on intercept	−.04(.33)	.8986	−1.69 (.75)	.0249
1^st^ grade RAN numeral RT on intercept	−.55(.31)	.0780		
1^st^ grade RAN letter RT on intercept			−1.66(.70)	.0183
Intelligence on slope	−.11(.07)	.1249	.40(.18)	.0282
In-class attentive behavior on slope	−.34(.08)	.0001	.13(.20)	.5004
1^st^ grade central executive on slope	−.24(.09)	.0069	.24(.23)	.2959
1^st^ grade phonological memory span on slope	−.03(.08)	.6798	.09(.20)	.6707
1^st^ grade visuospatial memory span on slope	.06(.07)	.4169	.04(.19)	.8448
1^st^ grade RAN numeral RT on slope	.00(.07)	.9712		
1^st^ grade RAN letter RT on slope			−.49(.17)	.0041
**Fifth Grade Working Memory and RAN RT Predictors of Fifth Grade Achievement**				
5^th^ grade central executive on intercept	−.14 (.15)	.3250	.96(.74)	.1934
5^th^ grade phonological memory span on intercept	.14 (.15)	.3518	.41(.75)	.5856
5^th^ grade visuospatial memory span on intercept	.28 (.13)	.0346	.81(.65)	.2126
5^th^ grade RAN numeral RT on intercept	.22 (.12)	.0618		
5^th^ grade RAN letter RT on intercept			−1.52(.58)	.0087

Note: Negative girl vs. boy on intercept estimates mean girls have lower scores in fifth grade. Positive girl vs. boy on slope estimates mean that the sex difference is smaller in earlier grades. RAN = rapid automatized naming; RT = reaction time (sec).

#### Numerical operations

The top portion of Table 3 indicates that girls answered, on average, 2.31 fewer problems correctly than did boys in fifth grade. The girl vs. boy on slope effect indicates that the difference found in fifth grade is significantly smaller in earlier grades. For instance, the estimated gap in fourth grade is 1.95 problems [i.e., −2.31+(1*.36)], 1.59 problems in third grade [i.e., −2.31+(2*.36)], and so forth. The results also indicate that independent of this sex difference children who scored higher on the intelligence (p = .0063) and central executive (p = .0007) measures in first grade and were rated by their second to fourth grade teachers as more focused and organized in the classroom (p = .0001) scored higher than their peers on the Numerical Operations test at the end of fifth grade.

The negative predictor on slope effects indicate that the importance of these competencies are lower in earlier grades, or stated differently are increasingly important as mathematical content becomes more complex across grades. First grade visuospatial memory was not related to fifth grade achievement (p = .8986), but there was a trend for speed of numeral processing (p = .0780); children who were faster at encoding and articulating Arabic numerals in first grade had a small advantage over their slower peers in fifth grade. Critically, the bottom portion of Table 3 indicates that children with higher visuospatial memory scores in fifth grade had higher same-grade Numerical Operations scores than did their peers with lower visuospatial scores (p = .0346), controlling for first grade visuospatial memory, first and fifth grade central executive scores, as well as all other predictors in the model. In contrast, fifth grade central executive scores did not predict fifth grade Numerical Operations scores (p = .3250), once the contributions of first grade central executive scores and other variables in the model were controlled.

The model shown in Table 3 was then rerun, replacing the total visuospatial memory score with the individual Block Recall and Mazes Memory scores. Neither of these variables, as measured in first grade, predicted fifth grade Numerical Operations scores (ps>.9070) or across grade changes in these scores (ps>.5129). There was a trend for fifth grade scores on Mazes Memory to predict fifth grade Numerical Operations scores, ß = .23, p = .0661, but Block Recall scores did not (p = .5036).

The presentation format for Block Recall is more similar to that of the central executive and phonological memory tasks than is the Mazes Memory format. One possibility then is that covarying central executive and phonological memory scores might suppress the potential relation between Block Recall and Numerical Operations scores due to similarity in presentation method. To assess this possibility, the model was rerun, dropping first and fifth grade central executive and phonological memory scores and their interactions with grade. In the resulting model, neither first grade Mazes Memory nor Block Recall scores were related to Numerical Operations scores (ps>.4776), but there was a trend for fifth grade Mazes Memory to predict fifth grade Numerical Operations scores, ß = .23, p = .071; Block Recall remained a non-significant predictor (p = . 4760).

#### Word reading

There was no sex difference in word reading achievement in fifth grade, but the significant girl vs. boy on slope effect indicates that girls had an advantage over boys in third grade and earlier. Independent of this sex difference a striking finding is that with the exception of intelligence, none of the predictors of Numerical Operations scores predicted Word Reading scores (Table 3). Children with longer phonological memory spans in first grade (p = .0433) and faster speed of letter encoding and articulation (p = .0183) had higher Word Reading scores at the end of fifth grade. The negative effect for first grade visuospatial memory on fifth grade Word Reading may be a spurious estimate due to the correlations between this variable and the central executive and phonological memory variables; dropping these variables reduced the visuospatial effect to non-significance (p = .2457).

The RAN letter on slope interaction (p = .0041) indicates that letter processing speed is a stronger predictor of individual differences in word reading fluency in earlier than later grades. Finally, children with faster letter processing speeds in fifth grade were more fluent word readers in fifth grade than were their peers with slow letter processing, above and beyond the influence of first grade letter processing speed (p = .0087).

## Discussion

The current study contributes to our understanding of developmental changes in the working memory system and to the relation between these changes and children’s emerging mathematical competencies, with a focus on visuospatial memory. Our finding that the competencies encompassed by the central executive, as assessed in first grade, predicted mathematics achievement throughout the elementary school years confirms previous findings [1]–[2], [5]–[6], [45], and thus we do not further discuss these results. Similarly, we do not consider further our findings that more intelligent children and children rated by their teachers as more attentive and organized in the classroom have an advantage over their peers in mathematics achievement, as these relations have also been previously reported (e.g., [42]–[43]).

### Working Memory Development

Our findings are consistent with the well-established pattern of age-related improvements in working memory performance [38], [55], and suggest that rate of improvement may differ across the three core components of the working memory system. Regarding these differences, it is useful to place our findings in the context of Gathercole et al.’s [38] cross-sectional study of more than 700 children. Specifically, we examined their results for the age groups that corresponded to the ages at our first and fifth grade assessments and for the same measures. Gathercole et al. found gains from 1.2 to 1.3 SDs for the corresponding central executive measures, 1.4 to 1.7 for the visuospatial measures, and 0.7 to 1.2 for the phonological measures. The magnitude of the gains from their cross-sectional comparison is consistent with our longitudinal findings for phonological memory (Table 1), but somewhat smaller than our findings for the central executive and visuospatial memory. The overall pattern, however, of relatively larger gains in visuospatial memory than gains in the capacity of the central executive, followed by comparatively smaller gains in phonological memory is the same across our longitudinal and their cross-sectional analyses. The pattern is also consistent with the shorter-term longitudinal studies noted in the introduction [16], [35].

Although much remains to be learned, gains in phonological and visuospatial memory can result from biologically-based increases in capacity, more efficient use of strategies to maintain or consolidate to-be-remembered information (e.g., rehearsal, chunking), or some combination [55]. The developmental gains in phonological memory disappeared once individual differences in the central executive were controlled, suggesting that much of this gain was related to improvements in rehearsal or other strategies to maintain the sounds in working memory rather than to biological changes in the capacity of this system [39], [55]. The gains in visuospatial memory were reduced by 63% with control of the central executive, phonological memory and their interaction, suggesting that strategy changes, such as phonological recoding of the visual stimuli, may have contributed to these gains [40], [56]. Nonetheless, the gains in visuospatial memory remained significant, even with further controls for processing speed, intelligence, and in-class attentive behavior, especially for Mazes Memory.

The non-experimental method does not allow for strong inferences, but the possibility that some aspects of the basic biological capacity of visual attention, spatial memory or related processes continue to develop from seven to eleven years of age should be considered [57]–[58]. Indeed, recent experimental evidence suggests that gains in visuospatial memory in particular may involve biologically-based increases in capacity [56].

### Visuospatial Memory and Mathematics

Whatever the mechanisms, children who showed the largest first-to-fifth grade gains in visuospatial memory had a mathematics achievement advantage over their peers at the end of fifth grade, controlling for other factors. This pattern combined with no relation between visuospatial memory and mathematics achievement in first grade suggests that visuospatial abilities become increasingly important to mathematics learning across grades, with one potential caveat. Our result suggest that it is not school entry visuospatial memory, as it is with central executive competencies, but degree of increase in this memory during elementary school. The implication is that individual differences in the either the rate of growth in visuospatial capacity or in the ability to strategically use these systems during mathematical problem solving (e.g., using diagrams to help solve word problems) must be considered with the study of and any interventions that target the spatial-mathematics relation [26].

Even though we did not find a relation between visuospatial memory and early mathematics achievement, broadly assessed, some other studies have found relations between some visuospatial abilities and performance on specific mathematical cognition tasks in young children [17], [23], [59]–[60]. These previous studies did not simultaneously control for intelligence, the central executive, processing speed, and in-class attentive behavior as we did here, although many of them controlled for a subset of these competencies and still found a relation. Gunderson et al. [23], for example, found a relation between skill at mentally rotating images and ease of learning the number line, controlling for verbal abilities. In short, although we did not find evidence for an early relation between visuospatial memory and mathematics, use of a broad mathematics achievement measure may have obscured more specific relations.

Our results for fifth grade Numerical Operations scores are consistent with Meyer et al.’s [24] cross-sectional finding that the central executive and phonological memory predicted second graders’ scores on this same achievement test, whereas the central executive and visuospatial memory predicted third graders’ scores. However, they used Block Recall as the visuospatial measure, which was not significant in our study. As described in the introduction, Kyttälä and Lehto [5] found that a measure of simultaneous visuospatial memory predicted overall mathematics achievement, as found here, and was especially predictive of performance on orally presented arithmetical word problems, whereas the competencies assessed by Numerical Operations in fifth grade involves whole and rational number arithmetic problems solved with pencil-and-paper. Our measures did not allow for an assessment of their finding that sequential visuospatial memory predicted competence at solving multistep word problems with pencil-and-paper or that active visuospatial memory predicted performance on mathematics problems that included an explicit spatial component (e.g., geometry items).

In other words, much remains to be discovered in terms of which visuospatial competencies predict which mathematical competencies, and which ages these relations emerge [26]. Despite these unresolved issues, our control of prior achievement, prior working memory competencies, as well as intelligence, in-class attentive behavior, and processing speed provides strong evidence that visuospatial memory not only contributes to individual differences in mathematics achievement, its importance increases across grades. This conclusion is bolstered by the discriminative contrast of the results for Numerical Operations with those for Word Reading; specifically, that visuospatial memory did not predict word reading fluency in any grade. The lack of relation between visuospatial memory and word reading fluency is not surprising, given the Word Reading test does not have any obvious visuospatial components. At the same time, our results for word reading support previous findings regarding the importance of phonological memory and speed of letter (and presumably word; [61]) retrieval for reading achievement [49], [62]. The consistency of our findings with previous results indicate that our sample and measures are well suited for the study of individual difference in achievement and achievement growth and highlight that, with the exception of intelligence, there are substantial differences in the cognitive mechanisms that are engaged during mathematical learning and word reading.

### Conclusions

The study confirms that children’s school-entry intelligence and attentional and inhibitory control, as measured by central executive tests, as well as their in-class attentive behavior, as rated by their teachers, all make independent contributions to mathematics learning during the elementary school years. Visuospatial memory did not contribute to individual differences in mathematics achievement early in the elementary school years, once these other factors were controlled, but children who showed large first-to-fifth grade gains in visuospatial memory had an advantage in mathematics but not reading achievement at the end of elementary school. The pattern suggests that children who show above average capacity gains in visuospatial memory or gains in the ability to strategically use these cognitive systems for problem solving may have a long-term advantage in mathematics.
